# Combination of Cloxacillin and Essential Oil of *Melaleuca armillaris* as an Alternative Against *Staphylococcus aureus*

**DOI:** 10.3389/fvets.2018.00177

**Published:** 2018-08-02

**Authors:** Daniel Buldain, Andrea V. Buchamer, María L. Marchetti, Florencia Aliverti, Arnaldo Bandoni, Nora Mestorino

**Affiliations:** ^1^Laboratory of Pharmacological and Toxicological Studies (LEFyT), Faculty of Veterinary Science, Universidad Nacional de La Plata, La Plata, Argentina; ^2^National Scientific and Technical Research Council, Consejo Nacional de Investigaciones Científicas y Técnica, Buenos Aires, Argentina; ^3^Department of Pharmacognosy, Faculty of Pharmacy and Biochemistry, Consejo Nacional de Investigaciones Científicas y Técnica, Institute of Chemical and Drug Metabolism (IQUIMEFA), Universidad de Buenos Aires, Buenos Aires, Argentina

**Keywords:** *Staphyloccocus aureus*, *Melaleuca armillaris*, essential oil, cloxacillin, resistance, antimicrobial, MRSA, synergism

## Abstract

The emergence of resistance to antibiotics has been favored by abuse in the application of antimicrobials in human and animal medicine. Essential oils are a great resource to deal with this crisis. *Melaleuca armillaris* belongs to the family of Myrtaceae, rich in species with essential oils. Plant extracts has shown antimicrobial activity in many investigations. Cloxacillin (CLOX) is an antibiotic widely used in veterinary medicine against *Staphylococcus aureus*. Our aim was to assess pharmacodynamic interaction established by combining essential oil of *M. armillaris* (EO) with CLOX in search of a synergistic effect that maximizes the antibacterial activity against *S. aureus*. The EO was obtained by steam distillation and its composition was analyzed by a GC–FID–MS. The most abundant components in the EO were 1.8 cineole (72.3%), limonene (7.8%). and α-pinene (6%). We worked with wild type *S. aureus* strains (*n* = 3) isolated from Holstein cows, and *S. aureus* ATCC 29213 as the reference strain. The Minimum Inhibitory Concentration (MIC) of CLOX, EO and the combination was determined by microdilution in broth at pH 7.4; 6.5 and 5.0. The checkerboard method was applied to evaluate the interaction between CLOX and EO. The Fractional Inhibitory Concentration index (FIC) was established. From those combinations that yielded the lowest FIC values, we evaluated the index of antibacterial activity (E), established as the difference between the Log_10_ values of the number of viable bacteria at the initial (nt_0_) and at the end of the test (nt_24_). So, time-killing curves with CLOX and EO/CLOX combination at 0.5, 1, 2, 4, and 8 fold the MIC in broth at pH 7.4; 6.5 and 5.0 were prepared. We considered Bacteriostatic effect (*E* = 0) Bactericidal effect (*E* = −3) and Effect of virtual eradication of bacteria (*E* = −4). A clear synergic activity between the EO and the CLOX was demonstrated, which allows reducing the MIC of β-lactam against *S. aureus*. This interaction was favored by acidification of the medium, where lower concentrations of CLOX achieved a bactericidal effect, close to virtual eradication, in the presence of small amounts of EO.

## Introduction

Bovine mastitis is one of the most prevalent problem which affects dairy herd production worldwide, and antimicrobial therapy is the primary tool for the treatment. This disease is responsible for a negative effect in the economy of several countries, because of the decrease results in the production level and quality of milk. The most frequent pathogen which caused subclinical mastitis in dairy cows is *Staphylococcus aureus* (1). This microorganism is characterized by its capacity of select resistance against traditional antimicrobials and some virulence factors as its ability to form biofilms and to invade and survive inside epithelial cells (2).

The emergence of resistance to multiple antibiotics has been favored by abuse in the application of antimicrobials in several areas: medicine, veterinary, agriculture.

At present, the growing number of methicillin-resistant *S. aureus* (MRSA) strains isolated from both groups, humans, and animals, hazards the efficacy of traditional antimicrobial treatments. The excessive and irrational usage of antimicrobials favored the emergence of resistance to multiple antibiotic families.

It is probably that the introduction of antimicrobials resistant to β-lactamases in the 1960s led to the emergence of MRSA (3). In veterinary medicine literature, the isolation of MRSA in animals has been reported since 1975 (4). This has been generating a dissemination of resistant and multi-resistant strains transmitted between humans and animals (5).

Cloxacillin (CLOX) is a semisynthetic antimicrobial derivate of penicillin that resists being breakdown by the enzyme penicillinase. CLOX is an antibiotic widely used in veterinary medicine against *S. aureus* (1). It has bactericidal activity against β-lactamase producing *S. aureus*. However, the increasing occurrence of methicillin-resistance and the consequent failure in therapy induces the search of new therapeutic alternatives. An alternative to face the problem of bacterial resistance is the use of products derived from plant extracts. In the past, natural and phytochemical products were used in the treatment and prevention of infectious diseases. Plants naturally synthesize aromatic chemical compounds and secondary metabolites which serve as a defense mechanism against microbes (6). The biological activity of a medicinal plant is usually based on the presence of one or a set of chemical components located in the tissues of the plant. The compounds with antimicrobial activity are mostly present in essential oils, which can occur in one or more organs depending on the species (7). Essential oils turn out to be the final product of the secondary metabolism of aromatic plants (8).

Essential oils are an interesting kind of herbal extract composed by complex mixtures with high antimicrobial activities. It is useful to take advantage of this property and transfer it to the treatment of bacteria resistant to antibiotics. There are many works that establish the restoration of antimicrobial activity of antibiotics, which have diminished their effectiveness against microorganisms, when they are combined with essential oils (9–12).

The exploitation of essential oils in the prevention of bacterial resistance is promising because those are multi-components with different targets of action, compared to many conventional antimicrobial agents that only have a single target site. Depending on the composition of the essential oils, different mechanisms of action can be attributed, including damage to essential proteins of the pathogen, blocking of membrane enzymes with which the microorganisms can pump out the active principle, changes in the metabolism, permeabilization of their membranes. These oils contain wide ranges of polyphenols and terpenoids, which have strong binding affinity to different molecular structures such as membranes, due to their great lipophilicity, presenting a high potential to penetrate through cell walls and disorganize them, leading to leakage cellular content (13).

Several authors are researching about the application of essential oils as adjuvant to increase the effect of antimicrobials against bacterial species. This is a new concept with high potential. For example *Pelargonium graveolens* essential oil reduces the minimum effective dose of norfloxacin against *Bacillus cereus, Bacillus subtilis, Escherechia coli*, and *S. aureus* (10). Rodrígues et al. (11) reported that the essential oil of leaves of *Croton zehntneri* is able to enhance the activity of gentamicin by 42.8% against *Pseudomonas aeruginosa* by gaseous contact, which suggests that the oil has a potential to be used as an adjuvant in antimicrobial therapy (11). Gram positive bacteria as *S. aureus* are more susceptible to essential oils than Gram negative ones (14), because these last one have an outer layer with lipopolysaccharide that cover the peptidoglycan and limiting the diffusion of hydrophobic compounds through it (15).

The antimicrobial properties of essential oils promote scientific interest in the study of them as a new group of pharmacological compounds. They possess a huge chemical and structural variety, converting them into functionally versatile mixtures (9). Although their activity can be associated with the major components, all of them influence in the pharmacological effect. It is probably that the high concentration of the major compounds masks the effects of the others. However, in terms of antimicrobial activity, every isolated component shows less antibacterial effect than the complete essential oil (16). It has also been shown that the function of the main compounds is regulated by the minority molecules, contributing to the synergic effect (17). Considering the large number of chemical structures that make up the essential oils it is likely that antibacterial properties cannot be attributed to a single chemical compound, and therefore there could be several targets in the microbial cells where these would act resulting in an enhancing influence. That is why it is reasonable to study the essential oils as a whole and take advantage of the synergy of all the components (18).

*Melaleuca armillaris* (Soland. ex Gaertn.) Sm. is one of the most widely cultivated Melaleuca plants. It is commonly known as Honey Bracelet Myrtle and grows in the form of a large bush or as a small tree. Reports of the literature about *M. armillaris* essential oil (EO) remain scarce. Investigations by GC-MS of its EO revealed the presence of 1.8-cineole as the major component (19–21). Several activities have been determined for this EO. Rizk et al. (22) obtained positive results *in vivo* using this EO in the treatment against the parasitoid *Schistosoma mansoni*, responding to the oxidant activity generated by the pathogen. Inhibitory activity was also found, *in vitro*, against some bacterial species such as *B. subtilis, S. aureus, S. epidermidis, E. coli*, and *P. aeruginosa* (22, 23). The activities observed for the EO of *M. armillaris* in pure form allow supposing that the good results obtained by themselves can be increased in synergistic systems for the same purpose, such as the improvement of antibiotics to treat infections caused by strains resistant to conventional treatment. No bibliography has been found indicating the use of the essential oil of *M. armillaris* as an enhancer of antibiotics used in the treatment of microbial infections, particularly against strains of *S. aureus*. This pathogen is very resistant to antibiotics, and is responsible for high percentage persistent infections. The indiscriminate use of antimicrobials is generating microorganisms resistant to multiple drugs, becoming a serious problem for global health (24).

Our aim was to assess the pharmacodynamic interaction established by combining EO of *M. armillaris* with CLOX emulating an extracellular and intracellular pH conditions (pH 7.4, 6.5, and pH 5) in search of a synergistic effect that maximizes the activity of the antibiotic against *S. aureus*.

## Materials and methods

### Plant material and essential oil extraction

Leaves and herbaceous branches of *M. armillaris* were collected in the month of July in the outskirts of Coronel Brandsen town, near La Plata city, Buenos Aires, Argentina. This region is located at latitude 35°06′18.9″S and longitude 58°10′57.0″W, and the climate is warm and humid with very rainy winters. The plant material was deposited in the LPAG Herbarium of the Faculty of Agrarian and Forestry Sciences, UNLP (25).

Vegetal material collection and extraction of the EO were carried out in the month of July during the morning. The extraction of EO was done by steam distillation with a distillation equipment of 385 liters of capacity. We used 38.5 liters of water for steam distillation and the amount of leaves and herbaceous branches was 44.750 kg. The EO obtain was dried with anhydrous Sodium Chloride, filtered and stored in amber glass bottle in a refrigerator at 4°C until use.

### Analysis of composition

The EO was analyzed by a GC–FID–MS Agilent (Agilent Technologies, Santa Clara, CA, USA) 7890A/5975C, equipped with one injector (split ratio 1:100), connected by a flow splitter to two capillary columns (HPWAX and DB-1-MS, both 60 m × 0.25 mm with 0.25 μm of fixed phase). The polar column was connected to a FID, whereas the non-polar column was connected to a quadrupolar mass detector (HP 5975C; 70 eV). Helium was used as gas carrier, at 1.8 mL/min. The injector temperature was set at 250°C. Injection volume was 0.3 μL. The column temperature was programmed according to the following gradient: 100°C, increasing at 2°C/min to 240°C and kept constant for 15 min. FID temperature was 260°C, and temperatures for the transference line and the ionic source were set at 280 and 230°C, respectively. Mass range (m/z) was 40–500 Da. Data acquisition, processing and instrument control was performed using the Agilent ChemStation (Agilent Technologies, Santa Clara, CA, USA) software. The identification of the compounds was achieved by analyzing the retention indexes (relative to C8–C24 n-alkanes) obtained in both columns and compared with those of reference compounds identified in chemically well-known essential oils and from bibliography (26, 27). Additionally, each mass spectra obtained was compared to those from the literature libraries (26, 28, 29) and mass spectra obtained from reference compounds. Relative percentage contribution of the compounds was calculated from the FID response by a computerized integration assuming all of the responses factors were 1.

### Microorganism and antibiotic

The study was carried out using 3 wild strains of *S. aureus* and the *S. aureus* ATCC 29213 as reference strain for quality control. Wild strains were isolated from milk samples obtained of Holstein cows with subclinical mastitis. They were identified by colony morphology and routine biochemical tests, Gram stain, coagulase, and catalase test, development of β-hemolysis, glucose fermentation and growth in saline medium (7.5%).

A standard of CLOX, pontency 96.4% (Sigma-Aldrich, chemical Company, St. Louis, USA) was used to performed this assay.

### Minimum inhibitory concentration (MIC) of CLOX and EO of *M. armillaris*

To determine the MIC of CLOX and EO, we used 96-well polystyrene microtiter plates with Mueller Hinton broth (MHB) supplemented with 0.5% of Tween 80 (to enhance the EO dissolution). The broth was adjusted to pH 7.4, 6.5, or 5.0 with hydrochloric acid 1N (Anedra, Argentina), in order to emulate the conditions of pH to extracellular and intracellular level. The range of CLOX concentrations (applying a scheme of two-fold serial dilution) evaluated was from 256 to 0.007 μg/mL. The concentrations of EO tested were from 50 μL/mL to 0.1 μL/mL. In both cases each well was inoculated with a final bacterial concentration of 5 × 10^5^ CFU/mL. The suspension was adjusted to match 0.5 Mc Farland (1 × 10^8^ CFU/mL). The plates were incubated at 35°C for 18–24 h. The MIC was established as the lowest concentration which inhibits the bacterial growth. All determinations were carried out in triplicate and in all cases positive and negative controls were used with MHB containing 0.5% Tween 80.

### Minimum inhibitory concentration (MIC) of CLOX/*M. armillaris* EO combination

The MIC of the mixture was performed as previously described by CLOX and EO. The combinations of the antibiotic and the plant extract were prepared according to checkerboard technique (30) against the strains selected. The microtiter plate consisted of a row with two-fold serial dilution of EO and a column with two-fold serial dilution of CLOX (as MIC control). The intermediate wells presented EO/CLOX combinations. The results were interpreted as for MIC of individual antimicrobials, but considering this parameter as a mix. The bacterial inoculums of *S. aureus* wild type and the reference strain used was 5 × 10^5^ CFU/mL per well. Incubation was carried out at 35°C for 18–24 h. The fractional inhibitory concentration index (FIC) was determined according to the following equation:

$$\begin{array}{l} \frac{\left(\text{A}\right)}{\left(\text{MIC}\right)\text{a}}+\frac{\left(\text{B}\right)}{\left(\text{MIC}\right)\text{b}}=\text{FIC} \end{array}$$

Where (A): CLOX MIC in combination with EO; (B): EO MIC in combination with CLOX; (MIC)a and (MIC)b: MIC of the antimicrobial and EO alone, respectively. Synergism (S) was considered if FIC ≤ 0.5; partial or low synergism (PS) if 0.5 < FIC < 1; indifference or addition (I) if 1 ≤ FIC <2 and antagonism (A) when FIC ≥ 2.

### Time-killing curves and antibacterial activity index of the AE/CLOX combination

The antibacterial activity index (E) was evaluated from those combinations that showed the lowest FIC values, established as the MIC value of the mixture. For this purpose we carried out time-kill assays for different concentrations (0.5MIC; 1MIC; 2MIC; 4MIC; 8MIC) of EO, CLOX and the combination. For the last one, 1MIC correspond to the lower FIC value blending and the others (0.5MIC, 2MIC, 4MIC, 8MIC) keep the proportion of both compounds (diluting or concentrating as the case may be). We prepared 7 tubes in each case (considering control positive and negative) with 1 mL of final volume containing MHB 0.5 Tween 80, antimicrobial (or AE/CLOX combination) and the inoculums. All tubes had a bacterial concentration of approximately 5 × 10^5^ CFU/mL (except the negative control). They were incubated at 35°C and samples were obtained at 0, 2, 4, 8, 12, and 24 h in order to construct survival curves by plate count. Those were performed for all strains at pH 7.4; 6.5 and 5.0.

The E index was quantified as the difference between the Log_10_ values of the number of viable bacteria (CFU/mL) at initial time (nt-0) and at the end of the test (nt-24) according to the following equation: E = nt-24-nt-0. To evaluate E, three theoretical cut points were applied (31): (a) Bacteriostatic effect: *E* = 0; there are no changes in the value of nt-0; (b) Bactericidal effect: *E* = −3; there is a reduction of ≥3 log_10_ of nt-0 and (c) Effect of virtual eradication of bacteria: *E* = −4; there is a reduction of ≥4 Log_10_ (99.99%) respect to Log of nt-0. The results obtained were plotted using the GraphPad Prism 6 program in order to obtain E vs. Log_10_ (concentration of CLOX) curves. The assays were performed in triplicate and the wild strains were grouped obtaining an *n* = 3.

## Results

**Figure 1:**
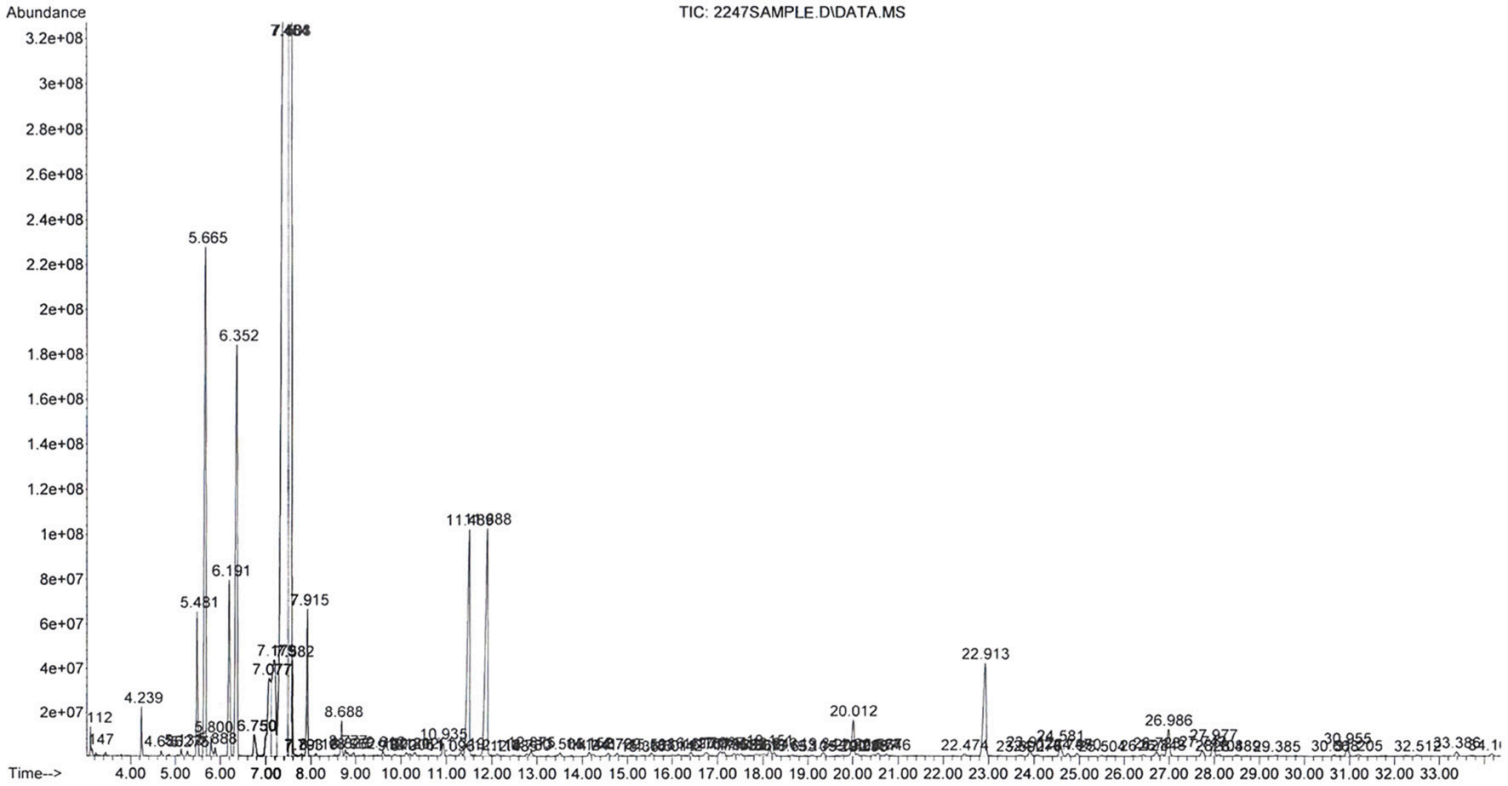
Mass spectra of all fractions *Melaleuca armillaris* pure essential oil by GC-FID-MS assay.

The whole distillation process lasted 5 h and 550 mL of essential oil were extracted. The analysis of composition revealed the presence of 1.8 cineole (72.3%), limonene (7.8%) and α-pinene (6%) as the most abundant components in the EO (Table 1, Figure 1).

**Table 1:** Percent composition of *M. armillaris* essential oil (EO).

**Compound**	**No polar**	**Polar**	**Area percentage**
α-Tujene	926	1036	1.5
α-Pinene	935	1043	6.0
Sabinen	968	1138	1.0
Myrcene	974	1170	2.2
β-Pinene	979	1133	2.2
α-Phellandrene	1005	1191	0.1
α-Terpinene	1012	1206	0.2
P-Cimene	1018	1286	1.4
1.8-Cineole	1022	1234	72.3
Limonene	1024	1221	7.8
Trans-β-Ocimene	1032	1260	0.2
γ-Terpinene	1047	1264	0.5
Terpinolene	1082	1305	0.1
δ-Terpineol	1150	1674	0.1
Terpinen-4-ol	1164	1614	1.4
α-Terpineol	1172	1705	1.4
Geranyl acetate	1359	1760	0.2
β-Caryophyllene	1417	1614	0.5
Aromandendrene	1437	1622	0.1
Geranyl isobutyrate	1496	1794	0.1
Cis-Calamenene	1508	1841	0.1
Oxi-Caryophyllene	1565	1989	0.1

The strains used in this study were identified as members of *S. aureus* specie after characterizing them biochemically, resulting in Gram positive, coagulase positive, catalase positive, β-hemolytic, glucose fermenting, and growth in saline (7.5%) strains.

The MIC of the CLOX obtained for *S. aureus* ATCC 29213 at pH 7.4 was 0.125 μg/mL, which is in accordance with the recommendations of the CLSI 2009 (32). The 3 wild strains (named SA13, SA96, and SA139) presented a MIC of 0.5 μg/mL at pH 7.4. In all cases, a decrease in this concentration was observed when acidifying the culture medium (Table 2).

**Table 2:** Cloxacillin MIC values obtained for the 4 strains at pH 7.4; 6.5 and 5.0.

**Strain**	**MIC pH 7.4 μg/mL**	**MIC pH 6.5 μg/mL**	**MIC pH 5.0 μg/mL**
ATCC 29213	0.125	0.062	0.031
SA 13	0.5	0.125	0.031
SA 96	0.5	0.125	0.031
SA 139	0.5	0.125	0.031

Respect the *M. armillaris* EO, for *S. aureus* ATCC 29213 the MIC was 25 μL/mL, while for the 3 wild strains it was 12.5 μL/mL. These values decreased slightly with the acidity of the culture medium (Table 3).

**Table 3:** MIC values of the EO at pH 7.4; 6.5 and 5.0.

**Strain**	**MIC pH 7.4 μL /mL**	**MIC pH 6.5 μL /mL**	**MIC pH 5.0 μL /mL**
ATCC 29213	25	25	12.5
SA 13	12.5	12.5	6.25
SA 96	12.5	12.5	6.25
SA 139	12.5	12.5	6.25

**Figure 2:**
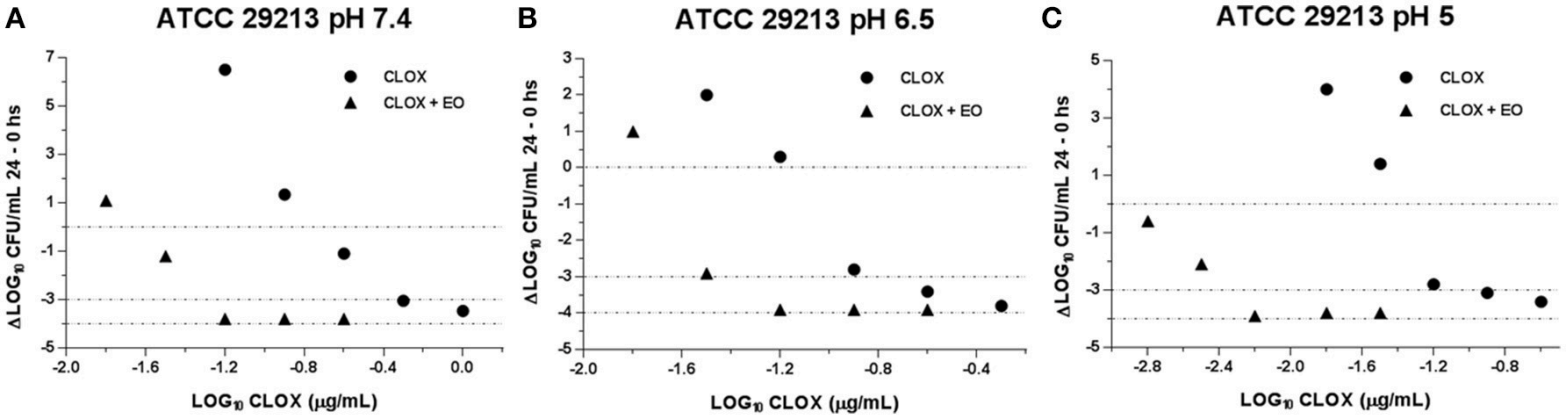
Graphic representation of the antibacterial effect (E: Δ Log CFU/mL 24–0 h) of Cloxacillin against *S. aureus* ATCC 29213 (*n* = 3) at pH 7.4 **(A)**; 6.5 **(B)** and 5.0 **(C)** in the presence and absence of EO.

**Figure 3:**
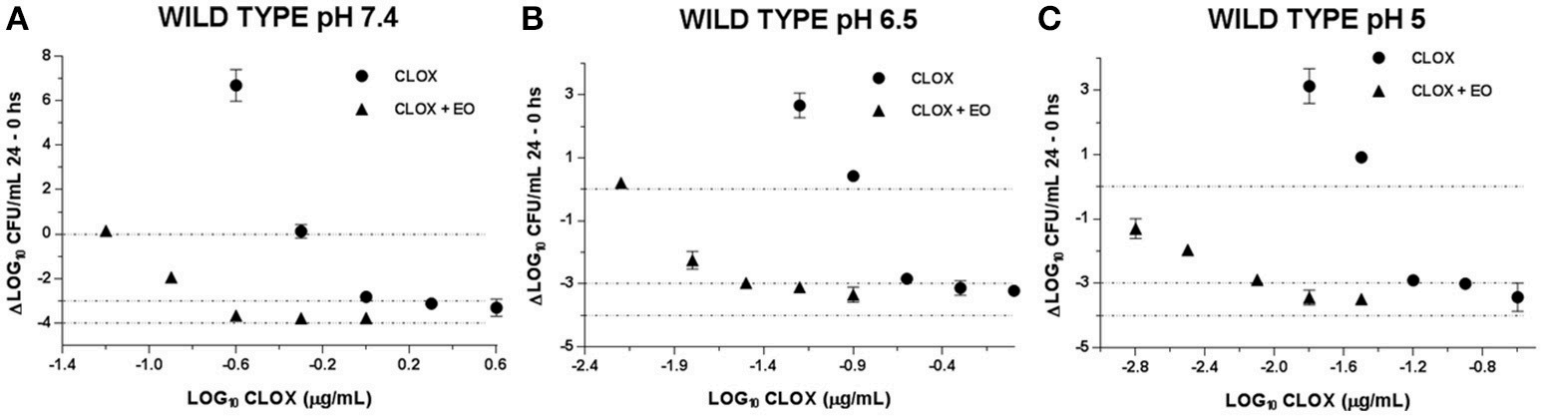
Graphic representation of the antibacterial effect (E: Δ Log CFU/mL 24–0 h) of Cloxacillin against WILD *S. aureus* strains (*n* = 3) at pH 7.4 **(A)**; 6.5 **(B)** and 5.0 **(C)** in the presence and absence of EO.

With the evaluation of the antibacterial activity index we could compare the incidence of EO in the CLOX activity, in addition to the effect caused by the pH variation. This is visualized in Figures 2, 3. It is observed how the acidification of the medium enhances the antimicrobial activity of CLOX against *S. aureus* (for the reference strain and the wild strains). Lower pH values requires a less amount of antibiotic to obtain an *E*-value lesser than −3 indicating a bactericidal effect. In the graphs it can be seen how the presence of the EO favors the action of the CLOX, since lower *E-*values are reached with lower concentrations of the antibiotic in comparison with the results obtained for CLOX alone. For the reference strain ATCC 29213, it was achieved a bactericidal effect very close to virtual eradication, since the *E*-values are close to −4. This was observed from concentrations of 2 the MIC of CLOX in the mixture, at the 3 pHs evaluated (Figure 2). In wild isolates the behavior was similar to that observed for the reference strain (Figure 3).

## Discussion

The chromatographic analysis of our EO revealed the presence of 1.8 cineole as major component (72.3%) and in lesser magnitude limonene (7.8%) and α-pinene (6.0%). Those are commonly present in effective antimicrobial essential oils. The 1.8 cineole is a monocyclic monoterpene with high antimicrobial activity. In several studies (19, 22, 33, 34), the main component reported was also 1.8 cineole, but this result disagree with other authors like Amri et al. (23) because the concentration of this one was markedly low (3.6%). There is evidence that the differences found in the concentration of the components in essential oils, extracted from different plants, would be affected by environmental conditions (35). The main compound of the mixture generally exerts the strongest activity, but can be influenced by the other molecules (36).

In our study, EO alone, without the addition of the antimicrobial, have demonstrated a strong antibacterial activity against *S. aureus*, this result agree with the research carried out by Amri et al. (23). The MIC against *S. aureus* ATCC 29213 was 25 μL/mL at pH 7.4 and it decreased 2-fold at pH 5. This result was also observed in wild strains.

A clear synergic effect was observed for the combination EO/CLOX, since FIC values obtained were ≤1 (Table 4). When we faced the EO/CLOX combination against the *S. aureus* strains, there was a great decreased in the concentration of antibiotic necessary to inhibit the bacterial growth. In EO/CLOX combination the bactericidal effect was maintained even when the pH of the broth was reduced from 7.4 to 5.0, as we have mentioned above when EO was studied without CLOX. And in the same way, when the culture medium was acidified, the concentration of CLOX, in the mixture EO/CLOX, was reduced even up to 10-fold to inhibit the microorganism. As we have mentioned in the introduction, there is still no published works where the pharmacological interaction between the *M. armillaris* EO and CLOX has been studied. In the bibliography another *Melaleuca* sp., *M. alternifolia* (tree of tea), has been widely investigated using the checkerboard technique against *S. aureus*. However, the results obtained when combining its EO with different antibiotics such as vancomycin (37), tobramycin (38), and ciprofloxacin (39) were indifference or antagonism effect.

**Table 4:** Fractional concentration indexes (FIC) obtained for the EO/CLOX combination under different pH conditions vs. individual MICs.

**Strain**	**pH 7.4**				**pH 6.5**				**pH 5.0**			
	**MIC EO μL/mL**	**MIC CLOX μg/mL**	**MIC EO/CLOX (μL/mL)/(μg/mL)**	**∑ FIC**	**MIC EO μL/mL**	**MIC CLOX μg/mL**	**MIC EO/CLOX (μL/mL)/(μg/mL)**	**∑ FIC**	**MIC EO μL/mL**	**MIC CLOX μg/mL**	**MIC EO/CLOX (μL/mL)/(μg/mL)**	**∑ FIC**
ATCC 29213	25	0.125	25/0.031	0.75	25	0.062	0.62/0.007	0.36	12.5	0.031	3.1/0.0035	0.36
13	12.5	0.5	6.25/0.125	0.62	12.5	0.125	12.5/0.015	1.12	6.25	0.031	3.1/0.0035	0.61
139	12.5	0.5	6.25/0.125	0.62	12.5	0.125	12.5/0.015	1.12	6.25	0.031	3.1/0.0035	0.61
96	12.5	0.5	6.25/0.125	0.62	12.5	0.125	12.5/0.015	1.12	6.25	0.031	3.1/0.0035	0.61

The combined activity of EO and CLOX, used in our study, was also evidenced in the bactericidal effect, established by the E index. The decrease in the initial bacterial inoculum -Log_10_ (CFU/mL)- in a factor of 3 in 24 h marked bactericidal activity observed in both for the CLOX alone and for the mixture. However, the concentration of the antimicrobial in the presence of EO was clearly lower and the *E*-value obtained was closer to the virtual eradication effect (*E* = −4) than the concentration needed when applying β-lactam alone. In a study conducted by Nascimento et al. (40) the essential oil of *Eremanthus erythropappus* was evaluated in combination with ampicillin (β-lactam antibiotic) against *S. aureus* achieving a synergistic bactericidal effect after 24-h incubation (40).

In conclusion, it was possible to reduce the concentration of the antibiotic needed to inhibit *S. aureus* by combining CLOX with EO *in vitro*. Considering that CLOX is an antimicrobial of the group of β-lactams with good activity against *S. aureus* and with wide use in veterinary medicine, the EO enhance CLOX antibacterial effect even when the intracellular pH were more acid than the extracellular medium. This is important for the treatment of intracellular infections where *S. aureus* is internalized inside of phagolysosomes because the probability of therapeutic success would be really increased. Our results suggest an increase of the susceptibility to β-lactams due to the acidic pH prevailing in the vacuoles where *S. aureus* live and prosper, which would be facilitated by the action of *M. armillaris* EO. The acid pH causes a conformational change of the target protein of action (PBP2a), increasing the affinity of its catalytic center for β-lactam (41). So the synergy we found between CLOX and EO in acid conditions could take place in the inner cell and have an important effect against *S. aureus* when it is refractory to immunological mechanisms. These findings become into a valuable alternative for the treatment of persistent staphylococcal infections. *M. armillaris* EO needs to be considered in the design of future formulations to evaluate *in vivo* effects, in order to maximize the efficacy of current and future antimicrobials.

## Author contributions

NM conceived and designed the experiments. DB performed all the experimental assay and statistical analysis. AVB, MM, and FA contributed with experimental assays. AB performed the EO quality assay. All authors contributed to the redaction, revision, and approved the final manuscript.

### Conflict of interest statement

The authors declare that the research was conducted in the absence of any commercial or financial relationships that could be construed as a potential conflict of interest.

## Abbreviations

CLOX: Cloxacillin
EO: *Melaleuca armillaris* essential oil
MHB: Mueller Hinton broth.
